# Development of endocytosis, degradative activity, and antigen processing capacity during GM-CSF driven differentiation of murine bone marrow

**DOI:** 10.1371/journal.pone.0196591

**Published:** 2018-05-10

**Authors:** Adesola C. Olatunde, Laura P. Abell, Ashley E. Landuyt, Elizabeth Hiltbold Schwartz

**Affiliations:** Department of Biological Sciences, Auburn University, Auburn, Alabama; Istituto Superiore di Sanità, ITALY

## Abstract

Dendritic cells (DC) are sentinels of the immune system, alerting and enlisting T cells to clear pathogenic threats. As such, numerous studies have demonstrated their effective uptake and proteolytic activities coupled with antigen processing and presentation functions. Yet, less is known about how these cellular mechanisms change and develop as myeloid cells progress from progenitor cells to more differentiated cell types such as DC. Thus, our study comparatively examined these functions at different stages of myeloid cell development driven by the GM-CSF. To measure these activities at different stages of development, GM-CSF driven bone marrow cells were sorted based on expression of Ly6C, CD115, and CD11c. This strategy enables isolation of cells representing five distinct myeloid cell types: Common Myeloid Progenitor (CMP), Granulocyte/Macrophage Progenitor (GMP), monocytes, monocyte-derived Macrophage/monocyte-derived Dendritic cell Precursors (moMac/moDP), and monocyte-derived DC (moDC). We observed significant differences in the uptake capacity, proteolysis, and antigen processing and presentation functions between these myeloid cell populations. CMP showed minimal uptake capacity with no detectable antigen processing and presenting function. The GMP population showed higher uptake capacity, modest proteolytic activity, and little T cell stimulatory function. In the monocyte population, the uptake capacity reached its peak, yet this cell type had minimal antigen processing and presentation function. Finally, moMac/moDP and moDC had a modestly decreased uptake capacity, high degradative capacity and strong antigen processing and presentation functions. These insights into when antigen processing and presentation function develop in myeloid cells during GM-CSF driven differentiation are crucial to the development of vaccines, allowing targeting of the most qualified cells as an ideal vaccine vehicles.

## Introduction

Dendritic cells (DC) are specialized immune cells that function in antigen uptake, processing and presentation, and induction of the adaptive immune response [1–3]. DC represent remarkable group of cells found in both lymphoid and non-lymphoid tissues under inflamed and/or steady state conditions. These cells have been classified into different subsets based on phenotypic and functional profiles [4, 5]. Phenotypically, expression of the integrin CD11c and high levels of MHC class II have been used to broadly identify DC. Subsets of DC are further separated based on expression of CD8, CD4, CD11b, and CD45R [6–8]. The functional attributes used in sub-setting DC include migration potential, antigen uptake capability, processing and presentation to the T cells [2, 9, 10]. Steady state DC, whose differentiation is dependent on Fms-like tyrosine kinase 3-ligand (Flt3L), represent conventional lymphoid resident or migratory DC [11–15]. The steady state DC that include both conventional DC (cDC) and plasmacytoid DC (pDC) differentiate from common DC precursor (CDP) and through an intermediate stage known as pre-DCs [16, 17]. Under inflammatory conditions however, monocyte-derived DC (moDC) development and differentiation is driven by Granulocyte-Macrophage Colony-Stimulating Factor (GM-CSF) [18–21].

Historically, it has been difficult to acquire sufficient numbers of DC directly *ex vivo* for functional analysis. DC are typically present in much lower numbers than lymphocytes in lymphoid organs and are of relatively low abundance in peripheral tissues as well [22]. Thus, for decades, GM-CSF has been a favorite cytokine used to generate large numbers of DC from mouse bone marrow *in vitro* [23–25]. Much of what we understand about the endocytic capacity, proteolytic activity, phagosomal maturation, and antigen processing and presenting function of GM-CSF-driven cells has come from studies on differentiated cells, DC and macrophages [26–30]. Thus, we know comparatively little about the developmental stage at which these functions develop. It is therefore important to investigate the development of these functions in order to identify the most qualified cells for therapeutic uses.

Recent studies have demonstrated the previously unrecognized heterogeneity of bone marrow cultured in GM-CSF [31, 32]. While the GM-CSF-driven culture method is known to generate a large population of CD11c^+^MHCII^+^ moDC, the relatively high frequency of monocyte-derived macrophages (moMac) in these cultures had not been appreciated [31]. These two cell types were distinguished based on the expression level of CD11c and MHC class II (moMac are MHCII^lo/int^, moDC are MHCII^hi^) [31]. Our recent study confirmed and extended these findings and identified an intermediate precursor cell, the monocyte-derived DC precursor (moDP), which shares many phenotypic characteristics with moMacs, but are distinguished by higher MHCII expression and ability to give rise to moDC [33]. Thus, this cell type is termed moDP.

To elucidate the functional capacity of each distinct myeloid cell population during GM-CSF driven differentiation, we have developed a sorting strategy that enables the isolation of five distinct myeloid cell populations based on the expression of Ly6C, CD115, and CD11c. The phenotypic profile of these developmental stages is as follows: common myeloid progenitor (CMP): Ly6C^-^CD115^-^CD11C^-^, granulocyte-macrophage progenitor (GMP): Ly6C^+^CD115^-^CD11C^-^, Monocytes: Ly6C^+^CD115^+^CD11C^-^, monocyte-derived macrophages/monocyte-derived DC precursors (moMac/moDP): Ly6C^-^CD115^+^CD11C^+^, and monocyte-derived DC (moDC): Ly6C^-^CD115^-^CD11C^+^ [33]. Thus, by isolating each of these populations, we set out to clearly define how the cellular uptake mechanisms, phagosomal acidification, and proteolytic activity change during myeloid cell development, and how these functional mechanisms impact the antigen processing and presentation function of these distinct myeloid cell populations along the developmental pathway.

## Materials and methods

### Mice

C57BL/6, OT-1 (C57BL/6-Tg(TcraTcrb)1100Mjb/J) and OT-II (B6.Cg-Tg(TcraTcrb)425Cbn/J) mice were obtained from Jackson Laboratory. T cells derived from OT-I mice recognize OVA_257-264_ peptide presented by MHC class I (H-2 K^b^), while OT-II mice recognize OVA_257-264_ peptide presented by MHC class II (I-A^b^). These mice serve as a source of large numbers of antigen-specific T cells for use in measuring presentation of that antigen (ovalbumin). All mice were housed under specific pathogen free conditions. All experiments were approved by the Auburn University Institutional Animal Care and Use Committee and were performed in accordance with the approved guidelines.

### Antibodies and reagents

Primary antibodies specific for murine CD11c (clone N418), CD206 (MMR) (clone C068C2), CD115 (CSF-1R) (clone AFS98), Dectin-1 (CLEC7A) (clone RH1), MHC class I and MCH class II (I-A^b^) (clone AF6-120) were from Biolegend (San Diego, CA). Ly6C (clone HK1.4) was purchased from Ebioscience and CD36 (clone CRF D2712), CD25 (clone PC61), CD4 (clone RM4-5) and CD8 (clone 53.6.7) were purchased from BD-Bioscience (San Jose, CA). DQ^™^ ovalbumin, OVA-Alexa 647, Dextran Alexa Fluor^®^ 488 (mw 10,000) Anionic Fixable, *Staphylococcus aureus* (wood strain without protein A) BioParticles^®^, Alexa Fluor^®^ 488 conjugate pHrodo^®^ Green *Escherichia coli* BioParticles^®^ were purchased from Molecular Probe (Eugene, Oregon). Nigericin sodium salt was purchased from Enzo Life sciences (Farmingdale, New York). Monensin sodium salt was purchased from Amresco (Solo, Ohio).

### Cells and sorting strategies

Myeloid cells were generated from C57BL/6 bone marrow (isolated from 8–24 week-old mice) as previously described [34]. In brief, bone marrow was harvested from mice and the red blood cells were lysed. The cells were cultured in RPMI media supplemented with 10% fetal calf serum (FCS), glutamine (2 mM) and 10 ng/mL granulocyte-macrophage colony-stimulating factor (GM-CSF). The cultured cells were sorted into 5 distinct myeloid cell populations: CMP, GMP, monocyte, moMac/moDP, and moDC using Beckman Coulter Moflo XDP High Speed Cell Sorter at the Auburn University Flow cytometry facility as described previously [33]. Briefly, cultured cells were harvested on either day 3 (to isolate early progenitor cells) or day 5 (to isolate more differentiated cell types) and stained with antibodies to CD115 (CSF-1R), Ly6C and CD11c. CMP, GMP, Monocyte, moMac/moDP, and moDC were isolated based on the phenotypic profile: Ly6C^-^CD115^-^CD11c^-^, Ly6C^+^CD115^-^CD11c^-^, Ly6C^+^CD115^+^CD11c^-^, Ly6C^-^CD115^+^CD11c^low^, and Ly6C^-^CD115^-^CD11c^+^, respectively. The five isolated cell types (from here on referred to as sorted populations) were re-suspended in growth media and were used for all experiments described below, except where indicated otherwise.

In order to differentiate between the moMac/moDP populations, day 5 cells were stained with antibodies to CD11b, CD11c, and MHC class II. Then, the CD11b^+^CD11c^+^ cells were isolated into MHCII^lo^, MHCII^int^, and MHCII^hi^ cell populations that correspond to moMac, moDP, and moDC.

### *Ex vivo* cell isolation

Freshly harvested bone marrow from C57BL/6 mice after lysing of the red blood cells were stained with antibodies to CD115, Ly6C and CD11c. These cells were sorted into CMP, GMP, and Monocytes based on the expression of these phenotypic markers and sorting strategies described above.

### Cell surface receptor staining

Bone marrow cells cultured in GM-SCF were harvested and stained with antibodies to CD36 PE, Dectin-1 PE, CD206 PE, CD115, Ly6C and CD11c from day 3 to 7. About 2 x 10^5^ cells were stained with each antibody at a final concentration of 2 μg/mL, and incubated at 4 °C for 30 min. Following the incubation period, cells were washed three times with ice cold FACS Buffer (PBS supplemented with 3% fatal Bovine serum). Unstained cells or cells stained with only one of the antibodies served as control in order to determine the level of auto fluorescence, compensate for fluorescence spillover, and set the gating boundary. The expression of these receptors was measured using flow cytometry.

### Phagocytosis/Macropinocytosis assay

Cells from each sorted cell population (2 x 10^5^ cells/well) were seeded in a 96 well plates. Dextran (0.5 mg/mL) or BioParticles (ratio of 2 particles to 1 cell) were added to the wells. The cells were incubated at either 37°C or 4°C (as a negative control) for 1 hr and the uptake of these endocytic tracers was measured using flow cytometry. The uptake capacity of each cell population was calculated by subtracting the mean fluorescence intensity (MFI) of samples incubated at 4°C from the MFI of samples incubated at 37°C for 1 hr.

### Phagosomal acidification/pH assay

Sorted cell populations were seeded at 2 x 10^5^ cells/well in 96 well plate, and were pulsed with 10 μL pHrodo^®^ Green *Escherichia coli* BioParticles^®^ for 15 min at 37°C, and washed twice by adding cold growth media, centrifuge at 1200rpm for 5 min and the supernatant was discarded. Cells were then resuspended in growth media and chased for additional 90 min at 37°C. At the end of the chase period, the cells were washed once with FACS buffer and analyzed using flow cytometry. The uptake of the BioParticles was measured using flow cytometry. To determine the actual pH of the pHrodo-containing phagosomes, pH calibration buffer containing 50 mM HEPES, 30 mM Ammonium Acetate, 10 μM nigericin, and 10 μM monensin was used. The pH of the calibration buffer was adjusted with HCl to create a standard curve of varying pHs, ranging from pH 2.5 to pH 7.5. After the 90 min chased period, cells from each sorted population were washed with FACS wash buffer and incubated with the pH calibration buffer for 15 min at 37°C and the samples were analyzed using flow cytometry. A pH standard curve was generated for each population by plotting the MFI of the pHrodo against the pH values. The standard curve equations generated from these graphs were used to predict the actual pH of the pHrodo-containing phagosome in each sorted population.

### Proteolysis assay

The proteolytic capacity of each cell population was determined by incubating 2 x10^5^ cells from each sorted population with 10 μg/mL DQ^™^ ovalbumin (DQ-OVA) for 15 min at 37°C. After the initial incubation, cells were washed twice with cold growth media (as described above) and chased for additional time points. Degradation of DQ-OVA (increasing fluorescence) was measured using flow cytometry. To control for differences in uptake capacity, sorted cell populations were incubated with both DQ-OVA and OVA-Alexa 647 for 1 hr. DQ-OVA fluorescence was then measured specifically in cells containing OVA-Alexa 647.

### In vitro T cell activation assay

CD8^+^ T cells were isolated from spleens of OT-I mice (C57BL/6-Tg(TcraTcrb)1100Mjb/J) and CD4^+^ T cells were isolated from spleens of OT-II mice (B6.Cg-Tg(TcraTcrb)425Cbn/J). About 6 x 10^7^ cells were labeled with 5 μM CFSE in Hanks’ Balanced Salt Solution at room temperature for 8 minutes while shaking gently every 2–3 mins. This was followed by adding 2 mL FCS for at least 1 min to stop the labelling, washed and counted before incubation with sorted populations. For OT-I cell stimulation, sorted cell populations were seeded in a 96 well plates and incubated with 300 μg/mL of OVA protein or with 10 nM OVA_257-264_ Peptide for 2 hrs followed with direct co-culture with CFSE labeled OT-I T cells. For OT-II cell stimulation, sorted cell populations were seeded in 96 well plates and incubated with either OVA protein (100 μg/mL) or OVA_323-339_ peptide (1 μM) for 2 hrs followed with direct co-culture with CFSE labeled OT-II T cells. About 5 x 10^4^ of sorted cell populations and 2.5 x 10^5^ T cells at a ratio of 1:5 was used for this experiment. T cell proliferation and activation was measured by flow cytometry after day 4 or 5 for OT-I and OT-II T cells, respectively.

### Flow cytometry

Where indicated, cells were stained with appropriate fluorophore-conjugated antibody in ice-cold FACS wash buffer (PBS + 3% FCS) on ice for 30 min. This was followed by washing twice with FACS wash buffer, centrifugation at 1200rpm for 5 min. Prior to acquisition, cells were resuspended in FACS wash buffer and filter through 35 μm. Unstained cells or cells stained with only one fluorophore were used to set the gates. In experiments where cells were loaded with fluorescence tracers, untreated samples serve as control and the gates were set on these untreated samples to determine the baseline fluorescence, and to set the compensation in that channel. Also, the forward and side scatter plots were used to exclude debris and doublets. All data were collected on an ACCURI C6 Flow cytometer (BD Biosciences) and analyzed with FlowJo software (Tree Star).

### Statistical analysis

The statistical analysis was performed using either GraphPad Prism 6 or Statistical Analysis System (SAS) software. All graphs were plotted in GraphPad Prism 6. The effect of treatment between different cell populations was analyzed using one-way analysis of variance (ANOVA), the Tukey multiple comparison PROC GLM procedure. Where indicated, treatments were considered significantly different at p< 0.05.

## Results

### Expression of uptake receptors during GM-CSF driven differentiation

**Fig 1:**
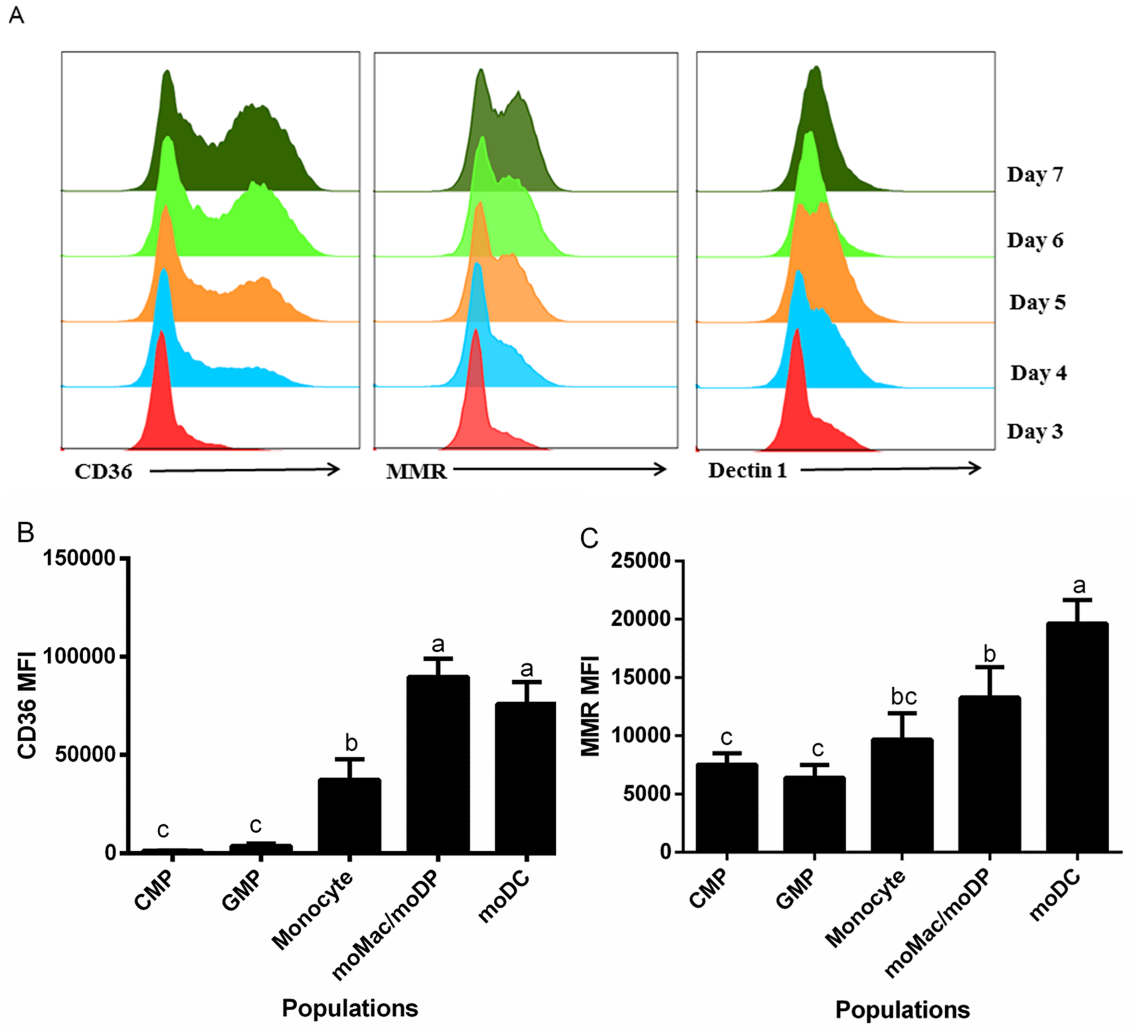
Differential expression of uptake receptors during GM-CSF driven differentiation. Bone marrow was harvested from C57BL/6 mice and was cultured in GM-CSF and stained with antibodies to CD36, CD206, or Dectin-1. (A) Representative histograms showing the expression of CD36, CD206, and Dectin-1 from day 3 to 7. Data were compiled from at least three independent experiments showing mean fluorescence intensity (MFI) of (B) CD36 and (C) MMR expression in the five cell populations. Statistical analysis was conducted using one way ANOVA Tukey multiple comparison test. Letters over bars indicate statistically significant differences in means (p<0.05).

Myeloid cells are known to express a wide range of uptake receptors such as scavenger receptors [35, 36], mannose receptors, and Dectin-1 [37, 38]. These receptors play important roles in cell adhesion as well as optimizing uptake of different types of particles or antigens. However, the relative expression of these receptors at distinct stages of GM-CSF-driven cell development is not well defined. Thus, we measured the expression of these receptors during GM-CSF driven differentiation of murine bone marrow from days 3 through 7. The heterogeneous populations of bone marrow were stained with antibodies to scavenger receptor (CD36), mannose receptor (MMR, CD206) and Dectin-1 and expression was measured by flow cytometry. There was an increase in the expression of CD36 and CD206 from days three to seven of culture with the expression of these two receptors peaking on day seven (Fig 1A). The expression of Dectin-1 increased from day 3 to day 5, with a slightly decreased expression on day 6 and 7 (Fig 1A). This result suggested that CD36 and CD206 are expressed at later stages during myeloid cell development while Dectin-1 is most highly expressed earlier during development.

We then measured the expression of these receptors with respect to distinct developmental stages, based on Ly6C, CD115 and CD11c expression. Interestingly, moMac/moDP and moDC, which were the dominant cell types on day 6 and 7, had the highest expression of CD36; whereas, CMP and GMP, the most prevalent cell types on day 3, had low expression of CD36 (Fig 1B). These results are consistent with a previous study that found high expression of scavenger receptor in DC, and the expression of this receptor is up-regulated upon DC maturation [39]. In contrast, all of the cell types expressed CD206 at relatively the same level with the exception of moDC that had a slightly increased expression (Fig 1C). This result is consistent with another study that found high expression of CD206 in immature DC [40].

### Distinct uptake capacities exhibited during GM-CSF driven differentiation

**Fig 2:**
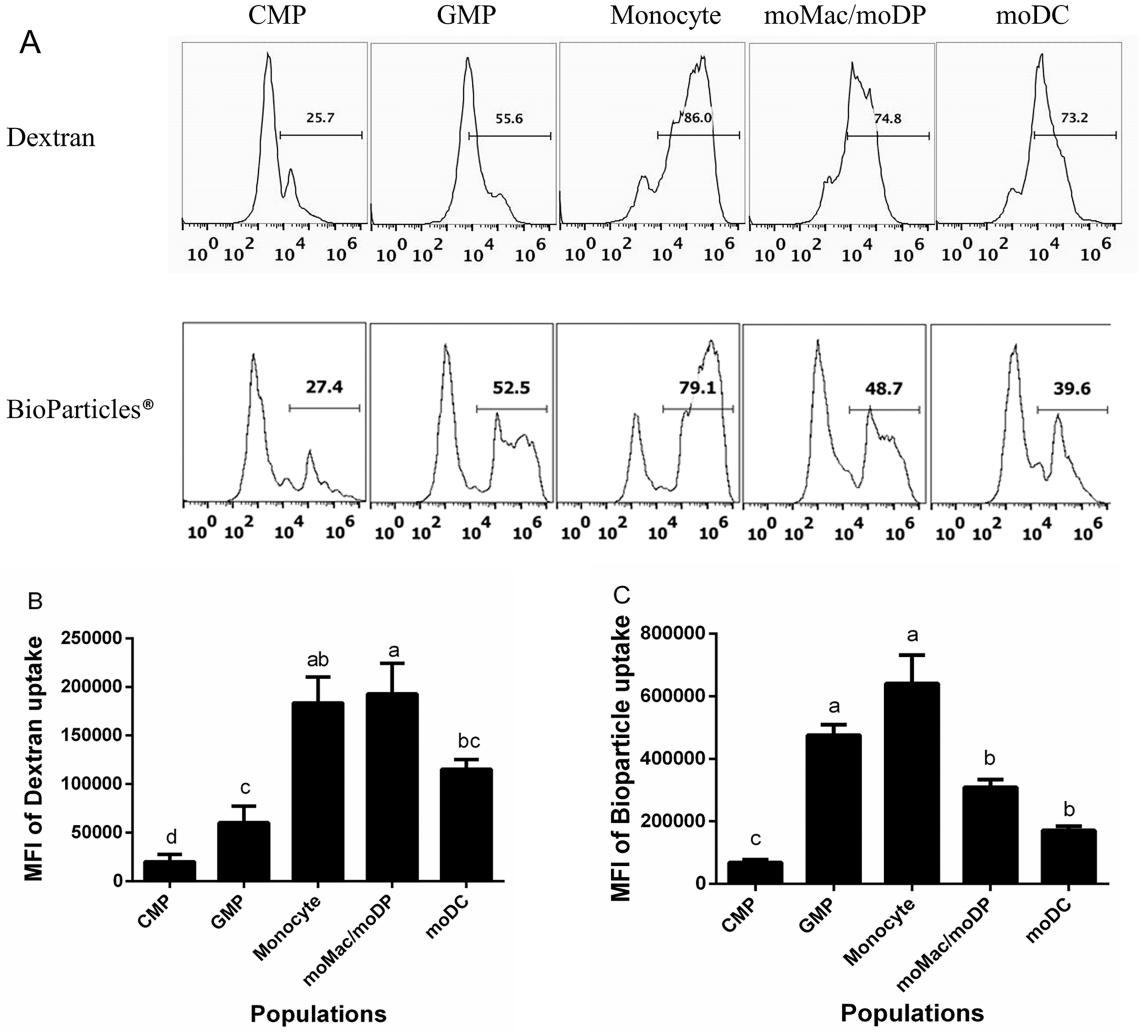
The uptake capacity of GM-CSF driven myeloid cells increased as cell progress in development and modestly decreased in more differentiated cell types. Sorted cell populations were fed 0.5 mg/mL Dextran Alexa Flour 488 (10,000 MW) or *S*. *aureus* BioParticle Alexa flour 594 conjugate for 1 h at 37°C (red), or at 4°C (blue) as negative control. The uptake of the fluorescent tracers was measured by flow cytometry. (A) Representative histograms of Dextran uptake (top panels) and BioParticle uptake (bottom panels) by the five isolated myeloid cell populations. Uptake of dextran (B) and bioparticles (C). Uptake values were calculated by subtracting the MFI of the ice control for each population from the MFI of that population incubated at 37°C. These data are compiled from at least four independent experiments. Statistical analysis was performed using SAS, one-way ANOVA Tukey’s multiple comparison tests. Letters over bars indicate statistically significant differences in means (p<0.05).

Immature DCs are known to utilize both macropinocytosis and phagocytosis as mechanisms of uptake [40–43]. However, endocytic capacity is down-regulated as dendritic cells undergo maturation [41]. What remains to be determined is how uptake capacity changes during development. Thus, to determine the uptake capacity of each myeloid cell population along the developmental spectrum, the sorted cells were fed one of two different endocytic tracers: Alexa-488 dextran (mw 10,000) as a measure of fluid-phase uptake or pinocytosis or Alexa-488 tagged BioParticles (*Staphylococcus aureus* wood strain without protein A), as a measure of phagocytosis. As illustrated by representative histograms (Fig 2A), CMPs demonstrated a low level of dextran uptake above the ice control, at a low level. Pinocytic activity increased only moderately in the GMP population, yet reached its peak in monocytes and moMac/moDP (Fig 2A and 2B). Pinocytic activity of moDC was slightly reduced compared to monocytes and moMac/moDP (Fig 2A and 2B).

The uptake of the larger cargo, BioParticles, followed a similar general trend as dextran uptake with some notable differences. CMP demonstrated virtually no phagocytic capacity (Fig 2A and 2C). Strikingly, GMP exhibited strong phagocytic activity, similar to that of monocytes (Fig 2A and 2C), and considerably stronger than their pinocytic activity (Fig 2B and 2C). Again, monocytes demonstrated the highest uptake capacity, with the highest phagocytic activity (Fig 2C). In contrast to dextran uptake, phagocytic activity also tended to decrease more significantly in moMac/moDP and moDC as demonstrated by decreased BioParticle uptake in these cell types (Fig 2C). These observed differences in uptake capacities of each myeloid cell type reflect significant functional diversity among these cell types.

### Phagosomal acidification increases progressively during GM-CSF-driven development

**Fig 3:**
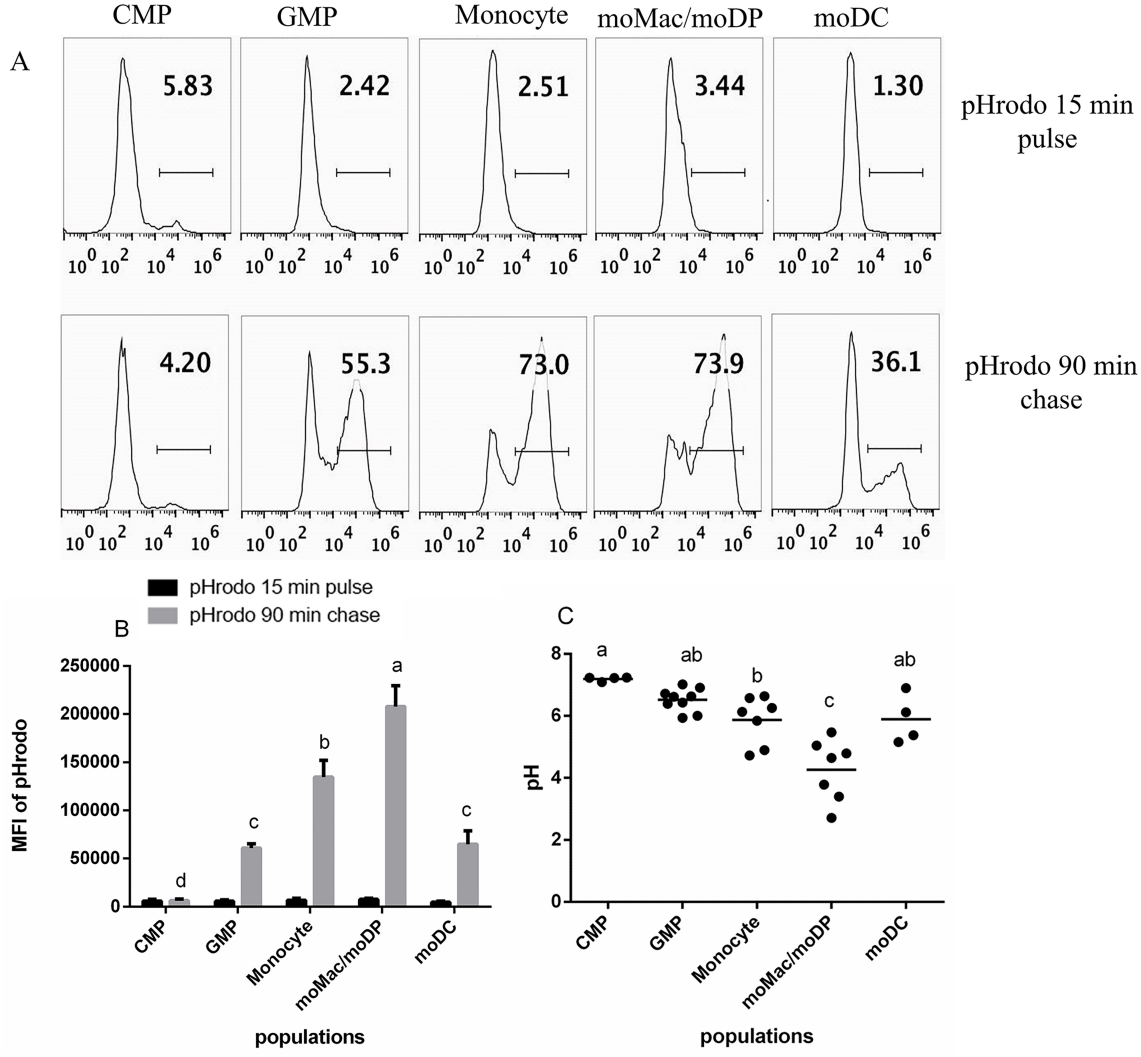
Phagosomal pH of each developmental stage. Sorted populations were pulsed with 10 μL pHrodo *E*. *coli* BioParticles for 15 min, washed and chased for 90 min. The uptake of the pHrodo *E*. *coli* BioParticles and the phagosomal acidification was measured by flow cytometry. (A) A representative histograms of pHrodo *E*. *coli* BioParticle uptake at 15 min (top) and phagosomal acidification at 90 min (bottom) in all the five isolated populations. (B) The MFI of pHrodo *E*. *coli* BioParticle uptake at 15 min and 90 min, in all the five isolated myeloid cell populations. Data are compiled from at least four independent experiments. (C) The phagosomal pH of each myeloid cell population was calculated vs a standard curve at varying pHs maintained in Hepes buffer containing nigericin and monensin. The statistical analysis was performed using SAS, one way ANOVA Tukey’s multiple comparison test. Letters over bars indicate statistically significant differences in means (p<0.05).

Acidification of the phagosome is one of the key events of phagosome maturation, and it is important for both antimicrobial and antigen processing functions [44, 45]. To assess the phagosomal pH of each myeloid cell population, we used a pulse-chase approach with pH-sensitive fluorescent particles (pHrodo Green *E*. *coli* BioParticles, Molecular Probes/Invitrogen). Cells were loaded with the pHrodo particles for 15 min, washed, and chased for additional 90 mins. This allowed us to track the acidification of a synchronized cohort of particles taken up during the short pulse period. Representative histograms of each cell population following the pulse of pHrodo *E*. *coli* BioParticles are presented in Fig 3A (top panels). Each population had a low background fluorescence indicating minimal acidification at this early time point (Fig 3A and 3B). After the 90 min chase period, phagosomal acidification became evident to different degrees in the five populations. Fluorescence was especially high in the monocyte and moMac/moDP populations (Fig 3A (bottom) and Fig 3B). CMP demonstrated the little to no phagosomal acidification (Fig 3A and 3B). The percent of pHrodo *E*. *coli* BioParticle-positive cells as well as the MFI of pHrodo gradually increased in GMP and monocytes and peaked in moMac/moDP population, decreasing substantially in moDC (Fig 3B).

Our previous studies indicated that the *Listeria monocytogenes*-containing-phagosomes in DC had a slightly elevated pH compared the same compartment in bone marrow derived macrophages [34]. To better understand phagosomal acidification across the developmental spectrum, we quantitatively compared the phagosomal pH of the five developmental stages driven by GM-CSF. To determine how fluorescence corresponded to actual pH values, standard curves were generated for each cell type in pH-normalized buffers using nigericin and monensin to equilibrate intracellular and extracellular pH. Phagosomal pH decreased progressively from the CMP stage (which was close to neutral pH 7.01) through the moMac/moDP stage which had the lowest pH at ~ pH 4.26. The pH of the moDC population was higher, ~ pH 5.89, which was very similar to monocytes (~5.87). GMP had an average pH of 6.52 (Fig 3C). Thus, these findings support our previous result indicating that moDC have a higher pH than moMac [34, 46]

### Proteolysis is a function exhibited later in the GM-CSF-driven developmental pathway

**Fig 4:**
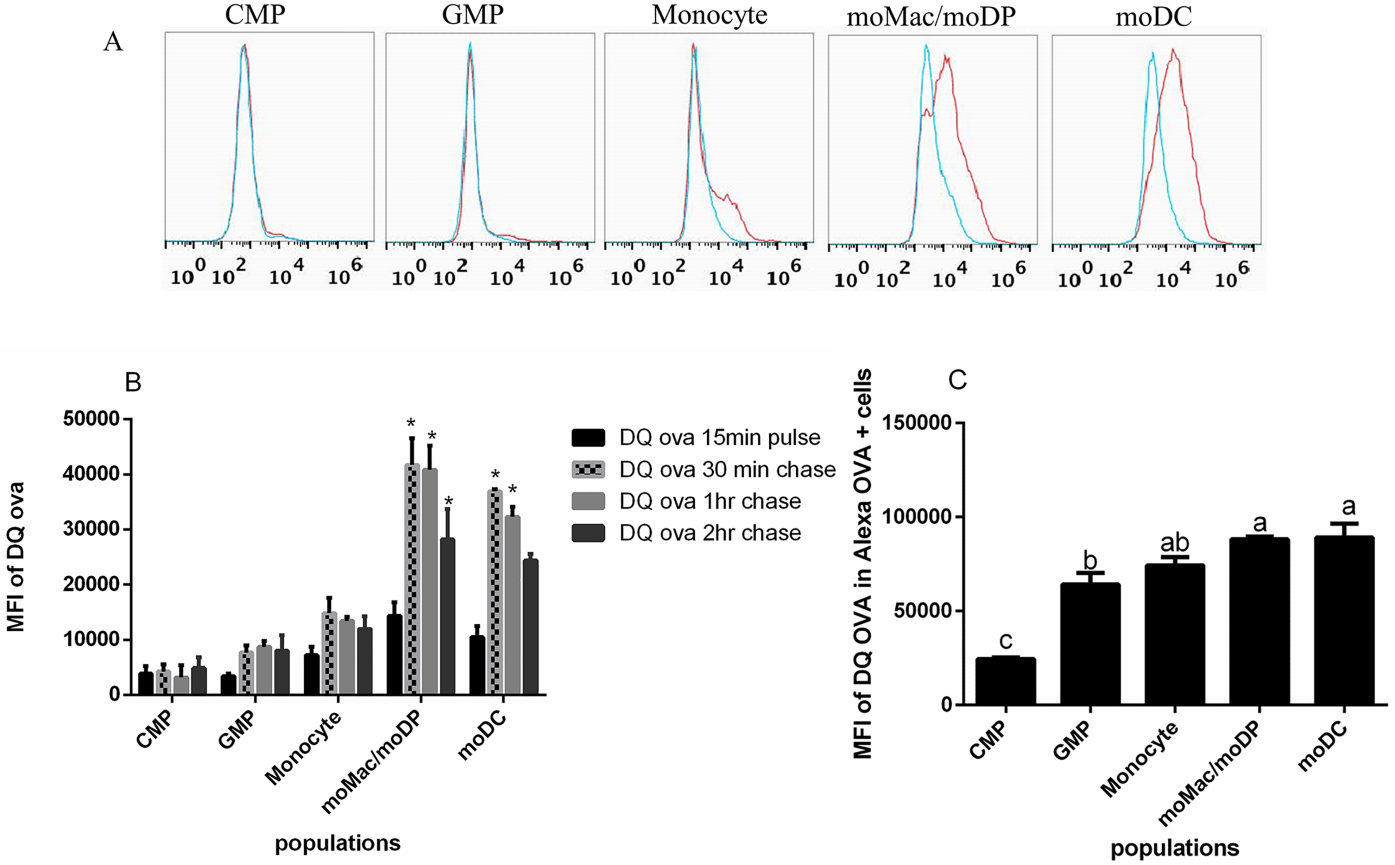
Proteolytic activity increases as myeloid cells become more differentiated. Sorted populations were pulsed with 10 μg/mL DQ^™^ ovalbumin (DQ-OVA) for 15 min, washed and chased for 30 min, 1hr or 2hr. Proteolysis (indicated by increased fluorescence) of DQ-OVA over time was measured by flow cytometry. (A) A representative histogram of DQ-OVA uptake at 15 min (blue) and degradation at 30 min (red). (B) The MFI of DQ-OVA at 15 min and degradation at 30 min, 1hr and 2hr in all the five isolated myeloid cell populations. (C) The MFI of DQ-OVA degradation only within OVA-Alexa positive cells measured at 1 hr post incubation. Compiled data come from a minimum of three independent experiments. Statistical analysis was conducted using one way ANOVA Tukey multiple comparison test. Asterisk indicate a p<0.05 when compared with the 15 min pulse.

To elucidate the proteolytic capacity of myeloid cells along the developmental spectrum, we utilized a fluorescence-quenched probe- DQ^™^ ovalbumin (DQ-OVA) that emits bright green fluorescence upon proteolysis. Each isolated cell population was pulsed with 10 μg/mL DQ-OVA for 15 min, washed and chased for additional 30 min, 1 hr, or 2 hr. As expected, there were no significant differences in the fluorescence of DQ-OVA across the populations after the initial 15 min pulse (Fig 4A and 4B) as this was likely too soon to observe degradation. After the 30 min chase, the moMac/moDP and moDC populations showed significant degradative activity evidence by increased fluorescence. This fluorescence was maintained at 1 hr chase, but decreased moderately by 2h, suggesting more complete degradation. In contrast, there was no significant increase in the proteolysis of DQ-OVA by the CMP and GMP populations (Fig 4A and 4B).

To control for any effect that the different uptake capacities of the five populations might have on the degradative measurements (Fig 2), we utilized an additional pH-insensitive tracer, OVA-Alexa 647, in combination with DQ-OVA to track the degradation only within the cells that had taken up the OVA-Alexa 647. In support of the previous result, we found that moMac/moDP and moDC had the highest degradative potential while GMP and Monocytes were slightly less degradative, and there was little to no proteolysis detected in CMP (Fig 4C). The data thus suggest that proteolytic activity is acquired after the CMP stage of myeloid cell development and peaks in the moMac/moDP and moDC stages.

### Antigen processing and presentation function is evident prior to myeloid cells differentiating into moDC

In order to assess the ability of each cell population to process and present antigen, we incubated them with OVA protein or peptide and analyzed their ability to stimulate proliferation and activation of naïve OVA-specific T cells (isolated from OT-1 (C57BL/6-Tg(TcraTcrb)1100Mjb/J) or OT-II (B6.Cg-Tg(TcraTcrb)425Cbn/J) mice). To measure proliferation, T cells were stained with CFSE prior to culture with each population and activation was measured based on CD25 expression.

**Fig 5:**
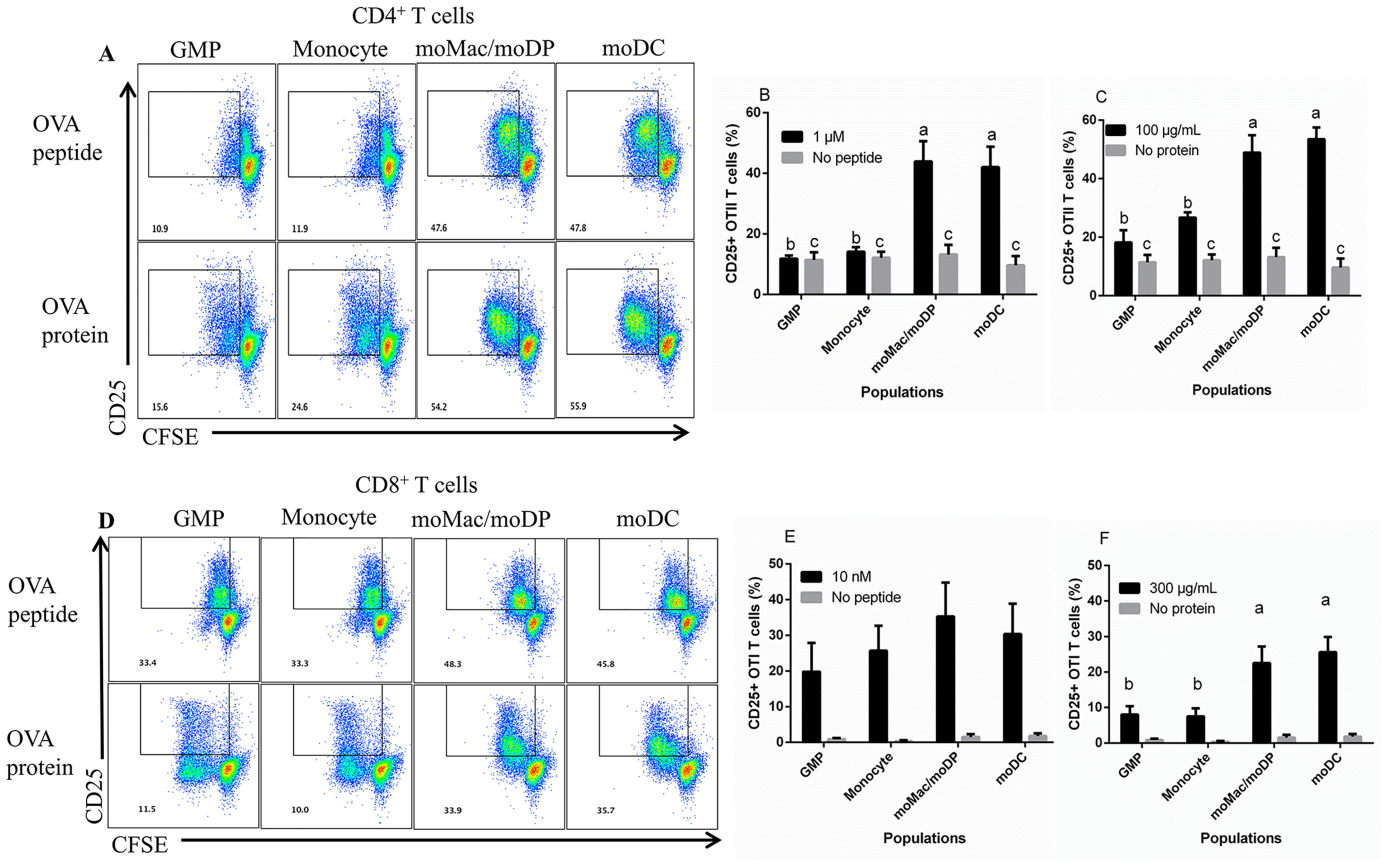
moMac/moDP and moDC exhibited both antigen processing and presentation functions. The sorted cell populations were incubated with different concentrations of OVA peptide or OVA protein and co-cultured with CFSE labeled T cells. (A) The dot plots illustrate CD4^+^ OT-II T cells stimulated with 1 μM of OVA_323-339_ (top) or 100 μg/mL of OVA protein (bottom). T cell proliferation and activation was measured using CFSE fluorescence dilution at day 5 by flow cytometry. (B) and (C) Compiled data of CD25 expression by OT-II T cells in the presence of OVA peptide (1 μM) or OVA protein (100 μg/mL), respectively. (D) The dot plots of CD8^+^ OT-I T cells stimulated with 10 nM of OVA_257-264_ (top) or 300 μg/mL of OVA protein (bottom). T cell proliferation and activation was measured at day 4 by flow cytometry. (E) and (F) CD25 expression by OT-I T cells in the presence of OVA peptide (10 nM) or OVA protein (300 μg/mL), respectively. Compiled data are derived from a minimum of 3 independent experiments. Statistical analysis was conducted using one-way ANOVA Tukey multiple comparison test. Letters over bars indicate statistically significant differences in means (p<0.05).

To assess antigen-presenting capacity, OVA peptides were used as the antigen source. GMP and monocyte populations were unable to stimulate robust OT-II proliferation and CD25 up-regulation when loaded with OVA_323-339_ compared to the no antigen control (Fig 5A and 5B; S2A, S2B and S2C Fig). However, GMP and monocytes were able to stimulate significant proliferation of OT-1 T cells when loaded with OVA_257-264_ (Fig 5D and 5E; S2D, S2E and S2F Fig). As a point of comparison, GMP and monocytes also had the lowest expression of MHC class II molecules, yet were similar in MHC class I expression (S1 Fig). Thus, the poor stimulatory capacity of GMP and monocytes to activate CD4^+^ T cells might be attributed to their lower expression of MHC class II molecules as well as reduced proteolytic activity (Fig 5B). In contrast, moMac/moDP and moDC were able to stimulate strong proliferation and activation of both OT-II and OT-I T cells when loaded with peptide (Fig 5A, 5B, 5D and 5E; S2A, S2B and S2C Fig).

Antigen processing capacity was tested using soluble OVA protein as the antigen source. GMP and monocytes were able to induce minimal activation and proliferation of OT-II cells when loaded with OVA protein (Fig 5A and 5C). Again, moMac/moDP and moDC induced the strongest OT-II proliferation and activation when loaded with OVA protein, indicating their strong antigen processing and presentation capacities (Fig 5D and 5F). Interestingly, when we assessed their cross presentation potential using OVA protein to stimulate OT-1 T cell activation, we observed that both moMac/moDP and moDC were potent at cross-presenting the protein antigen, while GMP and monocytes induced OVA-specific CD8^+^ T cell proliferation but induced lower level up regulation of CD25 (Fig 5D and 5F).

CMP induced little CD4^+^ or CD8^+^ T cell proliferation or CD25 expression in the presence of OVA protein or OVA peptide (data not shown). This observation was not surprising given that CMP had minimal uptake capacity, proteolytic activity and phagosomal acidification.

### The moMac and moDP population have similar functional capacities

**Fig 6:**
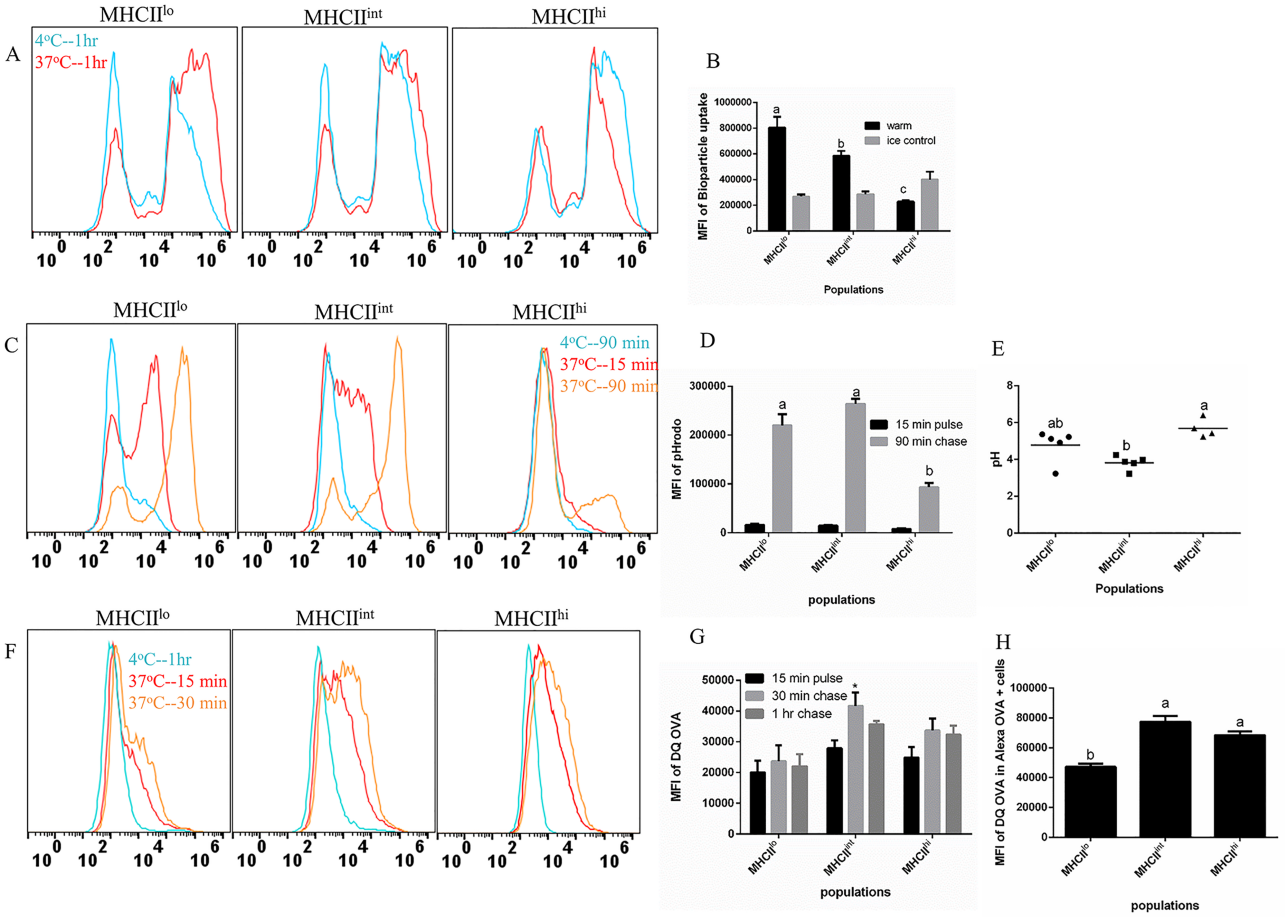
The MHCII^lo^ and MHCII^int^ cell populations are similar in uptake capacity and phagosomal acidification when compared with MHCII^hi^ cells. Cells were sorted into three populations on day 5 of culture: MHCII^lo^, MHCII^int^ and MHCII^hi^ after first gating on CD11c^+^, and CD11b^+^ cells. (A) Sorted cells were fed *S*. *aureus* BioParticle-Alexa flour 594 conjugate for 1 h. Representative histograms of BioParticle uptake at 4°C (blue) or 37°C (red). (B) Compiled MFI of BioParticle uptake from three experiments. (C) Representative histograms of cells pulsed with pHrodo *E*. *coli* BioParticles at 15 min (red) and chased for 90 min (brown) or at 4°C for 90 min (blue). (D) The compiled MFI of pHrodo *E*. *coli* BioParticle at 15 min and 90 min. (E) Calculated phagosomal pH of each cell population based on standard curves. (F) Representative histograms of cells pulsed with 10 μg/mL DQ-OVA for 15 min (red) and chased for 30 min (brown) or at 4°C for 1 hr (blue). (G) Compiled MFI of DQ-OVA at 15 min and degradation at 30 min and 1hr. (H) The compiled MFI of DQ-OVA degradation within OVA-Alexa positive cells measured at 1 hr post incubation. Statistical analysis was performed using SAS, one-way ANOVA Tukey’s multiple comparison test. Letters over bars indicate statistically significant differences in means (p<0.05).

We had previously determined that moMac/moDP and moDC (all CD11c^+^) could further be sub-divided based on the expression of CD11b and MHC class II (MHCII), with MHCII^lo^, MHCII^int^, and MHCII^hi^ cell types corresponding to moMac, moDP and moDC, respectively [33]. In order to examine the functional differences between the moMac, moDP and moDC populations, cells were isolated into three populations based on the expression of these molecules as previously described [33]. We observed that MHCII^lo^ population had the highest phagocytic capability, with a slightly lower BioParticle uptake by MHCII^int^, and the MHCII^hi^ cells had the least phagocytic activity (Fig 6A and 6B). The observed decrease in the uptake potential of MHCII^hi^ population especially when compared to the MHCII^int^ precursor cell, moDP, supports the concept that as DC become more differentiated toward maturation, they down-regulate their endocytic capacity [41].

In addition, we measured the phagosomal pH in each population and found that MHCII^lo^ and MHCII^int^ had highly increased fluorescence at 90 min at a level higher than the background fluorescence observed at 15 min after the initial incubation with pHrodo (Fig 6C and 6D). Interestingly, we found that MHCII^lo^ and MHCII^int^ had an average pH 4.76 and 3.82, respectively (Fig 6E), a pH comparable to that observed in moMac/moDP (pH 4.26) (Fig 3). However, MHCII^hi^ populations still demonstrated the highest phagosomal pH, with an average pH 5.68 (Fig 6E), a pH closer to that observed in moDC (Fig 3).

Finally, we assessed the proteolytic activity of these cells by utilizing DQ-OVA. There was a slight increase in the fluorescence of the DQ-OVA across all populations between the initial 15 min pulse and the 30 min chase (Fig 6F and 6G), with MHCII^int^ demonstrating the highest proteolysis at the 30 min chase period. However, when we utilized the OVA-Alexa 647 to control for differential levels of uptake, (examining only the cells that had taken up the OVA-Alexa 647), we observed that both MHCII^int^ and MHCII^hi^ had a slightly higher degradation potential when compared to the MHCII^lo^ population (Fig 6H). Taken together, these results indicate that the moMac population had the highest phagocytic activity, moderate pH and mild proteolysis while the moDP population was slightly less phagocytic and had the lowest phagosomal pH and highest degradative activity. Finally, the moDC population had greatly reduced phagocytosis, more neutral pH and moderate degradative activity.

### Functional capacity of early progenitors isolated directly ex vivo

**Fig 7:**
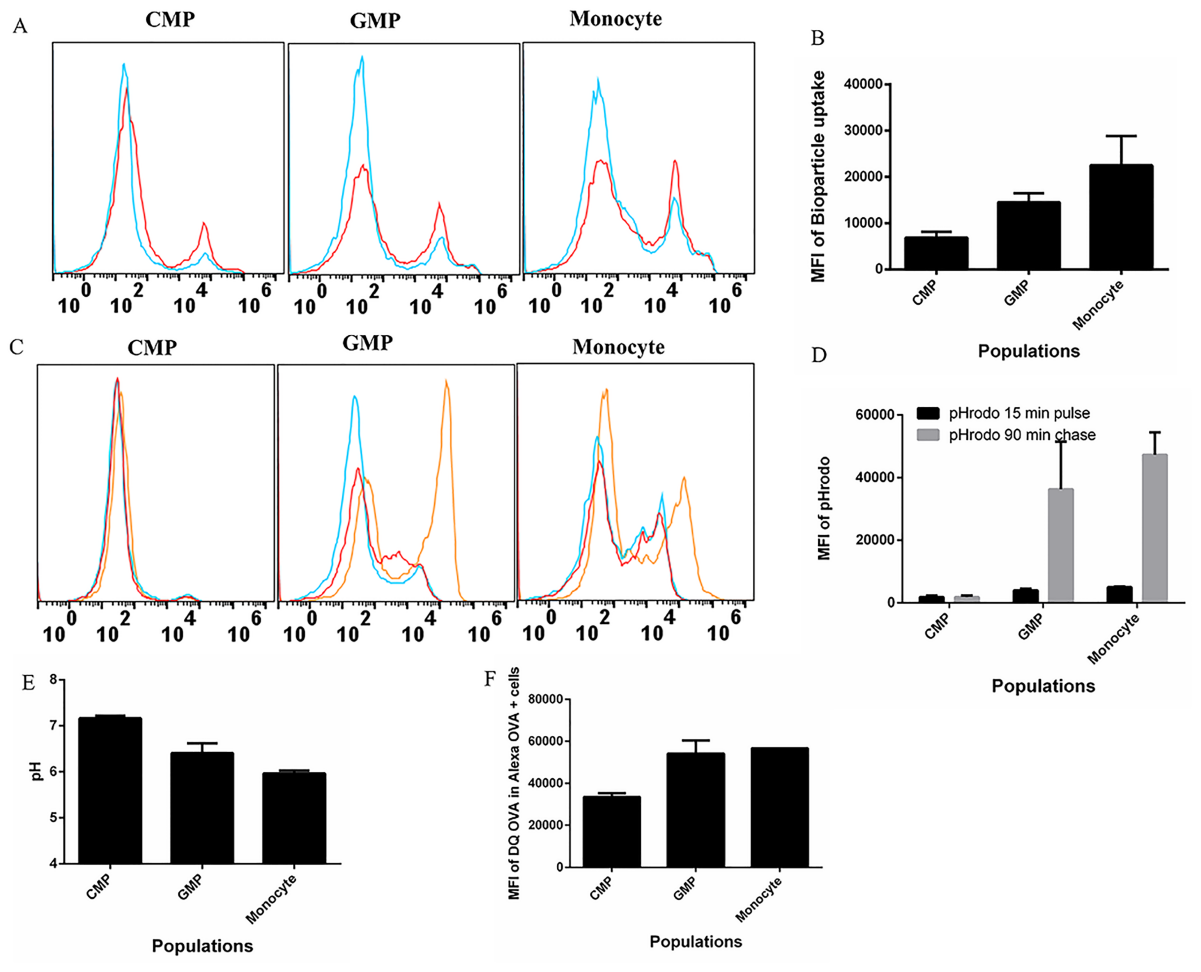
CMP, GMP and monocyte derived *ex-vivo* are similar in functionality to *in vitro* GM-CSF derived counterpart. Freshly harvested *ex-vivo* bone marrow cells were sorted into 3 populations has previously described. (A) Sorted cells were fed *E*. *coli* BioParticle-Alexa flour 594 conjugate for 1 h. Representative histograms of BioParticle uptake at 4°C (blue) or 37°C (red). (B) Compiled MFI of BioParticle uptake from three experiments. (C) Representative histograms of cells pulsed with pHrodo *E*. *coli* BioParticles at 15 min (red) and chased for 90 min (brown) or at 4°C for 90 min (blue). (D) The compiled MFI of pHrodo *E*. *coli* BioParticle at 15 min and 90 min. (E) Calculated phagosomal pH of each cell population based on standard curves. (F) The compiled MFI of DQ-OVA degradation within OVA-Alexa positive cells measured at 1 hr post incubation.

Finally, to ensure that our functional analyses were reflective of those observed *in vivo*, we examined the functional capacity of early progenitor cell types CMP, GMP, and monocytes freshly isolated from bone marrow. We found that CMP had the lowest uptake capacity, followed with increased uptake of Bioparticles in GMP, and the monocyte population had the highest uptake capacity (Fig 7A and 7B). Consistent with our *in vitro* studies (Fig 3), CMP isolated *ex vivo* had the highest phagosomal pH (pH ~7.14), with GMP having an average pH of 6.42, while monocytes had a slightly more acidic pH (pH ~5.92) (Fig 7C, 7D and 7E). Lastly, we observed that CMP had the least proteolytic capacity when compared with GMP (Fig 7F). Thus, we conclude that our *in vitro* cultured cells: CMP, GMP and monocytes closely resemble their *ex vivo* counterparts.

## Discussion

Most studies of DC function such as endocytosis, proteolysis, and antigen processing and presenting function have focused on fully differentiated DC. As a result, less is known about when these cellular mechanisms develop or how they change as myeloid cells progress from progenitor cells to more differentiated cell types. In this study, we have now determined that as the cells develop, they undergo significant changes in phenotypic and functional characteristics including: expression of uptake receptors, endocytic capacity, phagosomal pH, proteolytic activity, and antigen processing and presenting capacity.

CMPs, the earliest cell type examined demonstrated little endocytic or phagocytic activity, low proteolytic potential, and the highest phagosomal pH of all of the populations, at near neutral. CMPs are progenitor cells that have the ability to give rise to all the myeloid cells [47]. As such, CMPs have not yet developed all the necessary cellular machinery that could either enhance their antigen uptake capacity or degradative potential. In addition, CMPs had little ability to induce T cell proliferation or activation. CMPs showed the lowest response in all of the functional analyses in this study. However, this does not indicate that these cells are functionally incompetent. As such, a recent study illustrated the immune suppressive activity of CMP and GMP in the context of tumors as evidence in the ability of these cells to inhibit T cell proliferation [48]. Taken together, our study shows that CMPs are not potent at inducing T cell activation, but does not rule out a role for these cells in immune regulation.

GMP, the second stage along the GM-CSF-driven developmental spectrum, demonstrated high phagocytic capacity, moderate proteolysis and slightly more acidic phagosomal pH. Also, GMP demonstrated only slight antigen presentation function, evidenced in their ability to present OVA peptide to CD8^+^ T cells (Fig 5D). This observation might be attributed to the fact that innate immune cells constitutively express MHC class I molecules, potentially accounting for the ability of GMP to induce CD8^+^ T cell proliferation. However, GMPs were only able to induce CD8^+^ T cell proliferation without activation, as evidenced in low expression of CD25. In addition, GMP had lower expression of MHC class II, which could explain the inability of these cells to induce CD4^+^ T cell proliferation.

Monocytes had the highest phagocytic capability, and high expression of the uptake receptors when compared with CMP and GMP. The high uptake potential observed in monocytes was expected because these cells are known to be highly phagocytic, and they are endowed with a large repertoire of uptake receptors [49]. In addition, monocytes demonstrated high proteolytic capacity and low phagosomal pH, two functional properties that also support the antimicrobial activities of this cell type [50]. There have been conflicting results about the role of monocytes in antigen processing and presentation. For instance, a recent study demonstrated that Ly6C^+^ monocytes can cross-present cell-associated antigen to CD8^+^ T cells in a manner similar to DC [51], while another showed that monocytes are poor at presenting antigen [10]. In this study, we observed that within this population, antigen processing and presentation functions started to develop as evidenced in their ability to induce minimal CD4^+^ T cell proliferation when given OVA protein. Notably, monocytes were poor at presenting OVA peptide to naïve CD4^+^ T cells when compared with moMac/moDP and moDC (Fig 5A). Perhaps the peptide was less stable in this population. However, we observed that monocytes could cross present OVA protein to CD8^+^ T cells and induce T cell proliferation.

moMac/moDP includes cell types that phenotypically resemble macrophages as well as precursor cells, moDP, that can further differentiate to moDC [33]. Thus, some of the functional characteristics of the moMac/moDP cell population closely resemble moDC. First, we observed that moMac/moDP had high expression of uptake receptors and moderate uptake of BioParticles and dextran when compared with monocytes. In addition, the kinetics of proteolytic activities showed that moMac/moDP had high degradative capacity and the lowest phagosomal pH when compared with other cell types. Macrophages are known to have antimicrobial properties and are highly degradative, in line with their function in clearance of invading microbes [52].

While this is the first study examining the function of moDP, the functional characteristics of moMac have been more extensively studied. The moMac can acquire different functional attributes depending on the environmental cues, activation status, and inflammatory signals [53–55]. In addition, moMac can change their phenotype from a proinflammatory mediator to anti-inflammatory mediator and vice-versa, demonstrating their functional plasticity [56, 57]. To further determine the functional differences between the moMac and moDP, the mixed cell population was separated into three distinct populations based on the expression of MHCII. When we compared uptake capacity in MHCII^low^ and MHCII^int^ populations, corresponding to moMac and moDP respectively, we observed that the moMac cell population demonstrated higher uptake capacity than moDP, and moDC (MHCII^hi^ population) (Fig 6A). Although different moMac populations have been shown to differ to some extent in phagocytic capacity [53, 58] owing to the distinct array of uptake receptors they express [59], moMac generally have a higher uptake capacity when compared with DC [53]. This observation was consistent with our results.

Moreover, the phagosomal pH of different macrophage subtypes may also be distinct. For example, pro-inflammatory macrophages maintaining a pH close to neutral while the anti-inflammatory macrophages rapidly acidify their phagosomes [29]. When we examined the phagosome acidification in moMac and moDP, we observed that both cell populations had lower phagosomal pH when compared with moDC (MHCII^hi^ population) (Fig 6). The highly efficient phagosomal acidification observed in moMac/moDP could make this cell better suited at controlling microbes, a function that will need to be further examined. Finally, moDP had a slightly higher proteolytic activity when compared with moMac.

Intriguingly, we found that antigen processing and presenting functions develop before GM-CSF driven myeloid cells became fully differentiated moDC. moMac/moDP had a strong ability to induce CD4^+^ T cell proliferation and activation at a rate that is comparable with moDC. The ability of moMac/moDP to stimulate CD4^+^ T cell activation was greater than that observed in GM-Macs, a cell type highlighted by Helft et.al (similar to moMac in our study) [31]. Our data demonstrated that moMac/moDP is a unique mixture of cell types including those with similarity to GM-Macs, but also potent at priming naïve CD4^+^ T cells in a manner similar to moDC. This potent T cell activation capacity is likely mediated by the moDP population present in this complex population.

moDC functions are well characterized in the literature. It is well established that as DCs mature, they down-regulate their uptake mechanisms in favor of presenting the processed antigen via MHC on the cell surface [26, 41]. The observed decrease in uptake of BioParticles by moDC when compared with moDP (Fig 2C) could be a result of moDC being more differentiated and having less phagocytic activity. However, the uptake of dextran in both moMac/moDP and moDC reflects their ability to continuously take in bulk extracellular fluid, likely by macropinocytosis. In addition, moDC demonstrated a similar proteolytic activity and slightly higher phagosomal pH than moMac/moDP. These observed differences between moMac/moDP and moDC might be because moDC are more differentiated. In fact, several studies have indicated that DC have low degradative potential and high phagosomal pH, allowing for preservation of antigen for immune recognition [52, 60, 61]. Interestingly, phagosomal pH is known to decrease at least initially upon DC maturation, attributed to full assembly of vATPase on the mature DC phagosomal membranes [26]. Future studies in this arena should thus include an examination of vATPase assembly and activity in developing cells. Among all cell types examined in this study, we found that moDC were most potent at inducing both CD4^+^ and CD8^+^ T cell proliferation and activation, reaffirming their status as professional antigen presenting cells.

In summary, we have been able to systematically show the function of distinct myeloid cell populations during GM-CSF driven differentiation and how these functions change as the cells progress through their development. Importantly, we have been able to demonstrate in this study that antigen processing and presenting capability is a function that is acquired prior to the cells becoming moDC. This insight is essential because it could enhance the utilization of most qualified cells to function as antigen presenting cells in the design of vaccines.

## Supporting information

S1 Fig The expression of MHC class I and MHC class II molecules during GM-CSF driven differentiation. Sorted cells were stained with antibodies to Ly6C, CD115, and CD11c. (A) MFI of MHC class I and (B) MHC class II expression in the five cell populations.
(TIF)

S2 Fig Histogram depiction of T cell activation from Fig 5. (A) The histogram depicts CFSE fluorescence dilution in CD4^+^ OT-II T cells stimulated with 1 μM of OVA_323-339_ (top) or 100 μg/mL of OVA protein (bottom). (B) and (C) Compiled data of absolute number of OT-II T cells in the presence of OVA peptide (1 μM) or OVA protein (100 μg/mL), respectively. (D) The histogram of CFSE fluorescence dilution in CD8^+^ OT-I T cells stimulated with 10 nM of OVA_257-264_ (top) or 300 μg/mL of OVA protein (bottom). (E) and (F) The absolute number of OT-I T cells in the presence of OVA peptide (10 nM) or OVA protein (300 μg/mL), respectively.
(TIF)
