# Supplementary figures and images for: Development of endocytosis, degradative activity, and antigen processing capacity during GM-CSF driven differentiation of murine bone marrow
Source: PLoS One. 2018 May 10;13(5):e0196591. doi: 10.1371/journal.pone.0196591 (PMC5944997; doi:10.1371/journal.pone.0196591)

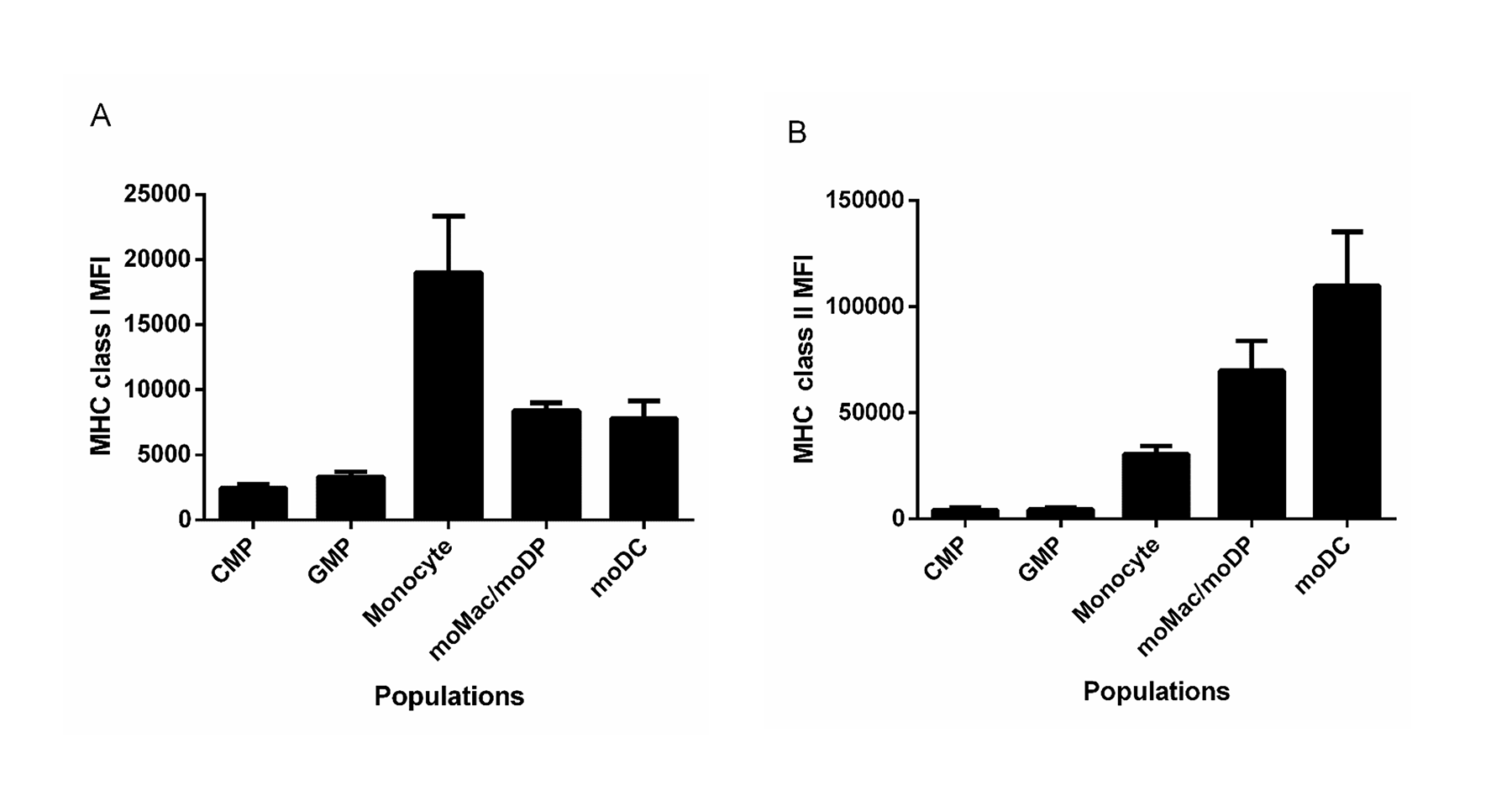

Supplement: S1 Fig — Sorted cells were stained with antibodies to Ly6C, CD115, and CD11c. (A) MFI of MHC class I and (B) MHC class II expression in the five cell populations. (TIF) [file pone.0196591.s001.tif]

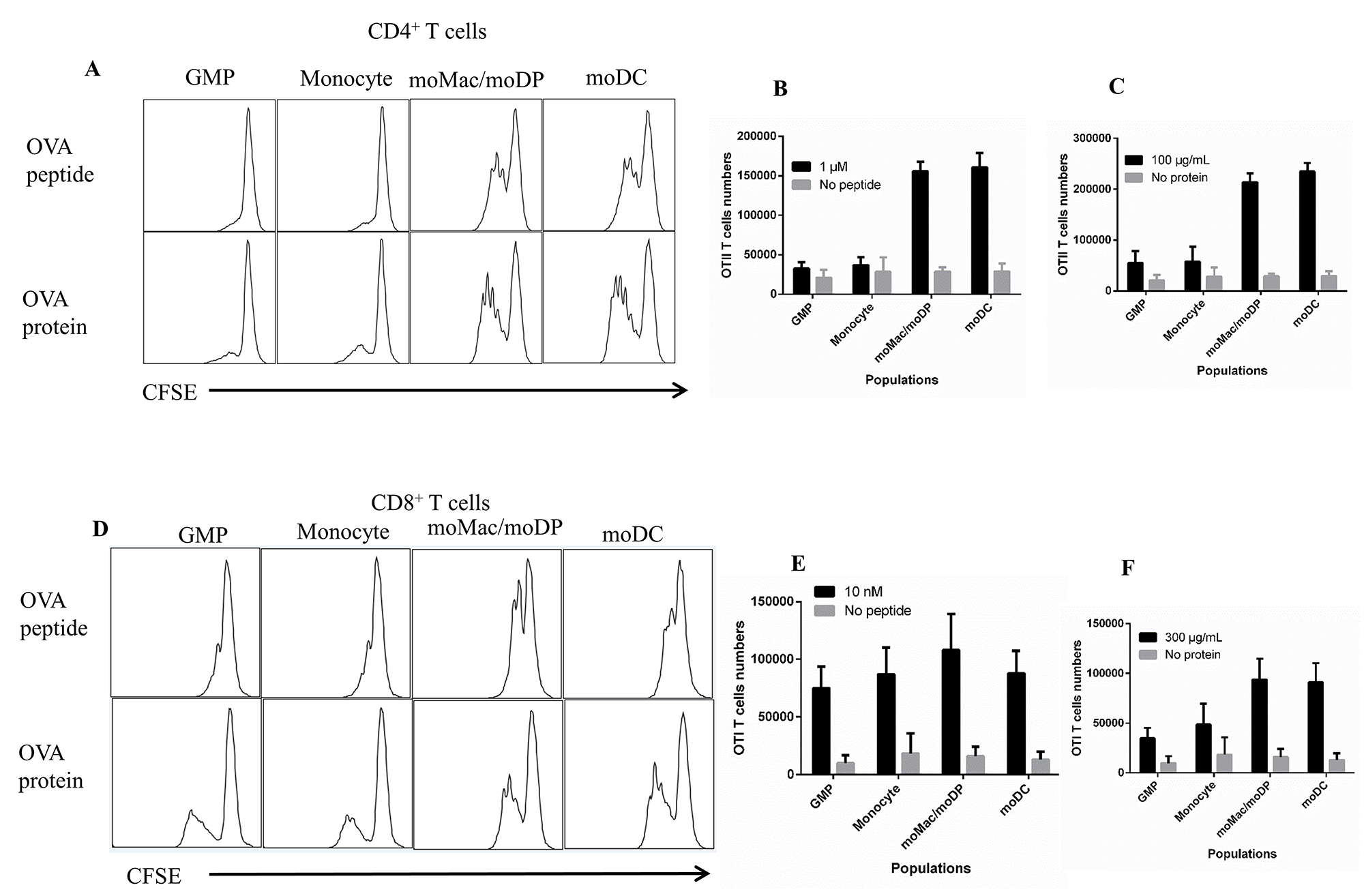

Supplement: S2 Fig — (A) The histogram depicts CFSE fluorescence dilution in CD4+ OT-II T cells stimulated with 1 μM of OVA323-339 (top) or 100 μg/mL of OVA protein (bottom). (B) and (C) Compiled data of absolute number of OT-II T cells in the presence of OVA peptide (1 μM) or OVA protein (100 μg/mL), respectively. (D) The histogram of CFSE fluorescence dilution in CD8+ OT-I T cells stimulated with 10 nM of OVA257-264 (top) or 300 μg/mL of OVA protein (bottom). (E) and (F) The absolute number of OT-I T cells in the presence of OVA peptide (10 nM) or OVA protein (300 μg/mL), respectively. (TIF) [file pone.0196591.s002.tif]
